# Influence of Membrane CD25 Stability on T Lymphocyte Activity: Implications for Immunoregulation

**DOI:** 10.1371/journal.pone.0007980

**Published:** 2009-11-24

**Authors:** Todd M. Brusko, Clive H. Wasserfall, Maigan A. Hulme, Roniel Cabrera, Desmond Schatz, Mark A. Atkinson

**Affiliations:** 1 Department of Pathology, University of Florida, Gainesville, Florida, United States of America; 2 Diabetes Center at the University of California San Francisco, San Francisco, California, United States of America; 3 Department of Pediatrics, University of Florida, Gainesville, Florida, United States of America; 4 Department of Medicine, University of Florida, Gainesville, Florida, United States of America; New York University School of Medicine, United States of America

## Abstract

**Background:**

CD25, a component of the IL-2 receptor, is important in T cell proliferation, activation induced cell death, as well as the actions of both regulatory (Treg) and effector (Teff) T cells. Recent genome wide association studies have implicated the CD25 locus as an important region for genetic susceptibility to a number of autoimmune disorders, with serum levels of soluble CD25 receptor (sCD25) serving as a potential phenotypic marker for this association. However, the functional impact of CD25 cleavage, as well as the influence of sCD25 on immunoregulatory activities, remain largely unknown and form the basis of this effort.

**Methodology/Principal Findings:**

The generation of sCD25 by Treg (CD4^+^CD25^+^) and Teff (CD4^+^CD25^−^) cells was examined during in vitro suppression assays, efforts that demonstrated constitutive and stable surface CD25 expression on Treg throughout the period of *in vitro* assessment. In contrast, Teff cells increased CD25 expression during the process of *in vitro* suppression, with supernatant sCD25 levels correlating to the amount of cellular proliferation. Interestingly, under serum-free conditions, Tregs partially lost their characteristic anergic and suppressive properties. sCD25 supplementation at physiological concentrations to serum free *in vitro* suppression assays reduced Teff proliferation without specifically influencing suppression. Indeed, sCD25 production within these cultures correlated with cell death.

**Conclusions/Significance:**

These results support the notion that sCD25 functions as both a surrogate marker of T cell activation as well as an indicator of subsequent cellular death. In addition, the role of CD25 in immunomodulation is likely dependent on the local inflammatory milieu, with molecules capable of modulating surface CD25 expression playing a key role in defining immune responsiveness.

## Introduction

Interleukin-2 (IL-2) has long been recognized for its role in promoting T cell proliferation *in vitro*, yet only recently has appreciation grown for its paradoxical role in maintaining peripheral tolerance *in vivo*. One key means by which IL-2 appears to promote tolerance involves its influence over CD4^+^CD25^+^ T cells, often termed regulatory T cells (Treg). In 1995, Sakaguchi and colleagues identified the α-chain of the IL-2 receptor (CD25) as a putative marker of functional Treg [1]. The high-affinity, trimeric IL-2 receptor complex consists of three major sub-units; the α-chain (designated CD25), the β-chain (CD122), and the common cytokine receptor γ-chain (CD132) [2]. These subunits, as well as other fragments of the molecule, appear to play important roles not only in immune physiology, but as markers of immune activity.

The concept that the expression level and affinity of cell surface receptors dictates cellular fate was introduced as the quantal theory by Kendall A Smith [3]. In activated T cells, signaling through the IL-2 receptor results in increased high-affinity receptor expression and eventual activation induced cell death (AICD) through up-regulation of Fas and FasL [4], [5]. It has been known since 1985 that production of the soluble form of CD25 (sCD25) associates with T cell activation *in vitro* [6]. In healthy individuals, sCD25 is present in serum at approximately 2 ng/ml, whereas increased serum sCD25 levels have been associated with lymphocyte activation in infection as well as in hematologic malignancies [7], [8]. More recently, polymorphisms in the *CD25* gene have been associated with autoimmune diseases such as type 1 diabetes, multiple sclerosis, and Graves' disease; with measurable differences in serum levels of the soluble form of the corresponding molecule (i.e., sCD25) [9]–[12]. These data point to, but do not specifically identify, a functional role for sCD25 separate from its value as a biomarker of activation in serum. Indeed, several key questions regarding sCD25 remain unanswered. These include those asking what biological actions result in the formation of sCD25, and which role (if any) does sCD25 play in modulating immune responsiveness?

While an alternative splice variant cannot be ruled out as contributing to the production of sCD25 [13], [14], it is more commonly thought that sCD25 results from a proteolytic cleavage event driven by a family of enzymes collectively referred to as “sheddases”. To date, at least three enzyme candidates have been reported to possess the capacity for cleaving CD25 including the endogenous enzymes elastase and matrix metalloproteinase-9 (MMP-9), as well as the environmental house dust mite allergen DerP1 [15]–[17]. While these enzymes have each been shown to be sufficient to elicit cleavage, the formation of sCD25 is often still observed in their absence or in the presence of enzymatic inhibitors. Thus, there may be additional unidentified-enzymes or overlapping enzymes responsible for sCD25 formation.

It has been suggested that sCD25 itself may bind IL-2 *in vivo*, preventing it from forming complexes with α2-macroglobulin and thus preserving its bioavailability for T cells [18]. The affinity of the monomeric soluble receptor for IL-2 is relatively low (K_d_ = 30 nM) [19]. This is comparable to the affinity of the monomeric membrane α-chain for IL-2 (K_d_ = 10^−8^ M), followed by that of the intermediate βγ complex (K_d_ = 10^−9^ M) and finally the high-affinity αβγ-trimeric complex (K_d_ = 10^−11^ M) [20]. An antagonistic role for sCD25 has also been suggested [21]. More recent studies have depicted sCD25 as an early inhibitor of IL-2 signaling in T cells that also enhances their proliferation in short and long term *in vitro* culture [12]. If this molecule is indeed participating in IL-2 signaling as suggested, then it may be possible to apply the quantal theory to this soluble receptor. sCD25 could then be expected to play multiple roles depending on the local environment in regards to IL-2 concentration, cellular activation state, and the amount of sCD25 present.

## Methods

### Objectives

We hypothesized that the control of membrane and soluble forms of CD25 on CD4^+^ T cells is critical for regulating immunity and tolerance, and that the presence of sCD25 and other factors in serum may be influencing *in vitro* assays. Herein, we describe efforts addressing these notions as well as our attempts to shed light on the aforementioned knowledge voids regarding sCD25 production and function. We sought to investigate the production of sCD25 during *in vitro* suppression assays as well as during PBMC activation. In addition, we explored the role of sCD25 during suppression and proliferation via addition of recombinant protein to *in vitro* assays for this activity. These efforts attempted to avoid interference from sCD25 present in supplemented serum by using serum free media. Taken together, we believe this effort provides valuable insights into the role of CD25 stability and sCD25 production on the process of immune regulation.

### Participants

Peripheral blood was obtained from normal healthy control subjects (11M/6F; median age 30.4, range 21.1 to 45.4 years) without a recent infection, allergic episode, or having a known autoimmune disorder.

### Description of Procedures or Investigations undertaken

#### *Staining and Flow Cytometry*

Cells were aliquoted (0.5–1×10^6^ in 100 µl per tube) along with each appropriate antibody, including anti-CD3 (clone HIT3a), anti-CD25 (M-A251), anti-CD4 (SK3), anti-CD127 (clone hIL-7R-M21), or isotype controls mouse IgG1 (X40), mouse IgG1 (MOPC-21), and mouse IgG2b [27]–[35] (BD Biosciences). FOXP3 staining was conducted with the anti-human FOXP3 (clone 206D) staining kit according to manufacturer recommendations (Biolegend). Flow cytometric analysis was conducted using FCS Express (De Novo Software) or FlowJo software (version 7.2.2, TreeStar).

#### *Cell Purification and Fluorescence-Activated Cell Sorting*

Cells used in functional suppression assays were separated into a T cell depleted accessory cell population (irradiated 3300 rads) and functional CD4^+^CD25^+^ Treg and CD4^+^CD25^−^ Teff cells. The untouched accessory cell population was produced by incubating an aliquot of blood (5 ml) with a T cell depletion antibody cocktail (RosetteSep; StemCell), followed by density gradient centrifugation (Cellgro) with Ficoll-hypaque solution (Amersham/GE Healthcare). CD4^+^ T cells were pre-purified from the remaining blood by negative selection using a CD4^+^ T cell enrichment cocktail (Stem Cell). Briefly, 50 µl of cocktail per 1 ml of whole blood was incubated for 20 min at 23°C, subjected to density gradient centrifugation, washed twice in PBS (Ca^++^ and Mg^++^ free) containing 0.1% BSA (Sigma-Aldrich), then this “untouched” CD4^+^ population underwent an additional high-speed FACS sorting procedure to yield CD4^+^CD25^Hi^ Treg cells and CD4^+^CD25^−^ Teff cells. For specified assays, the CD127 marker was also used during sorting to yield CD4^+^CD127^−/lo^CD25^+^ T cells, as these cells have been reported to contain a highly enriched FOXP3^+^ population [22]. Sorted Treg and Tconv cell populations were analyzed for purity following FACS with anti-human FOXP3 (Figure S1). PBMC for later proliferation experiments were isolated by density gradient centrifugation over Ficoll-Hypaque.

#### *Isolation of Purified Treg and Teff Cell Populations by FACS*

Pre-enriched CD4^+^ T cells were stained by the addition of anti-CD3 (clone HIT3a), anti-CD25 (M-A251), and anti-CD4 (SK3) antibodies at 5 µl/10^6^ cells. PBS containing 2% human AB serum was added for a final staining volume of 10^8^ CD4^+^ T cells/ml (30 min at 4°C), washed in PBS/0.1% BSA wash buffer, centrifuged (300 x g), and resuspended in wash buffer (3 ml) prior to high-speed cell sorting on a BD FACSVantage Cell Sorter. Sort gates for CD4^+^CD25^Hi^ T cells were set to optimize the yield and purity of FOXP3 expressing T cells within the CD4^+^CD25^+^ T cell fraction [23]. Where indicated, anti-CD127 ((clone hIL-7R-M21), BD Biosciences) was added to discriminate Treg from Teff cells. Purified CD4^+^CD25^HI^ and CD4^+^CD25^−^ T cell fractions were collected in sterile tissue culture tubes (BD Biosciences) containing cold (4°C) AIM V SFM (Invitrogen).

#### *Cell Culture*

Suppression assays were performed in AIM V complete SFM (Invitrogen) with the addition of freshly isolated autologous serum (heat inactivated at 56°C for 30 min), where indicated. PBMC proliferation assays took place in either RPMI 1640 (CellGro) supplemented with 5% human AB serum (Cellgro), or AIM V, XVIVO 15, or CTL Test serum free media. All assays were performed in U-bottom 96-well plates (Costar) and maintained at 37°C in 5% CO_2_.

#### *Suppression Assay Co-culture System*

CD4^+^CD25^Hi^ Treg cells were added in decreasing ratios (1∶0, 1∶1, ½∶1, and 0∶1) to a constant number of CD4^+^CD25^−^ Teff cells (5×10^3^ cells/well). A combination of 5 µg/ml soluble anti-CD3 (clone HIT3a) and 2.5 µg/ml soluble anti-CD28 (clone CD28.2; eBioscience,) provided proliferation stimulus for a 120 h culture period. 5×10^4^ irradiated (3300 rads) T cell depleted accessory cells were also added to each well in a total volume of 200 µl. One µCi of ^3^H-Thymidine (Amersham Biosciences) was added at 96 h for a final 16 h of culture to assess proliferation. Supernatants (20 µl/well) from triplicate wells were collected at 48 h and 96 h to assess sCD25 and cytokine production. Suppression was calculated by the reduction of ^3^H-thymidine incorporation using the following equation: Percent suppression  =  (1 - (mean CPM Treg+Teff)/(mean CPM Teff) ×100%). For FACS based suppression assays, proliferation was calculated by division index (DI), with suppression calculated as previously described [24]. For T cell subset proliferation assays, suppression assays were performed as above but with co-cultures containing either normal or irradiated Treg and Teff cell populations (3300 rads), or both.

#### *Fluorescent Cell Labeling*

To track proliferation of Treg and Teff cells individually, each cell population was labeled with either the “red” dye PKH26 (Sigma, final labeling concentration 2 µM) or “green” dye CFSE (Invitrogen, 1 µM), for Treg and Teff cells, respectively [24]. For cell labeling experiments, functional CD4^+^CD127^lo/−^CD25^+^ Treg and CD4^+^CD127^+^ Teff cell populations were obtained by FACS. Suppression experiments were conducted as described for ^3^H-thymidine based suppression assays, in the following combinations (Treg to Teff cell ratios 1∶0, 1∶1, 1/2∶1, 1/4∶1, 1/8∶1, 1/16∶1, 1/32∶1 and 0∶1; where 1 = 5×10^4^ cells). In addition, 5×10^5^ T cell depleted and irradiated (3300 rads) accessory cells were added. Cultures were run in triplicate and pooled prior to FACS analysis. At harvest, cells were washed in PBS containing 0.2% BSA, stained with CD4-APC (BD Biosciences), and analyzed by FACS for loss of cellular fluorescence in both proliferating CD4^+^ populations.

#### *PBMC Proliferation Assays*

Freshly isolated PBMC were plated at 10^5^ cells per well. Cells were left unstimulated or treated with PHA-L (Sigma-Aldrich) at 5 ug/ml, or α-CD3 and α-CD28 coated beads (Miltenyi) at a bead∶cell ratio of 1∶2. In addition, recombinant sCD25 was added at 2 and 20 ng/ml to duplicate wells for each condition. One µCi of ^3^H-Thymidine (Amersham Biosciences, Piscataway, NJ) was added at 96 h for a final 16 h of culture to assess proliferation. Supernatants were collected at 96 hours to measure sCD25 and nuclear matrix protein 41/7. Stimulation indices were determined by dividing the mean CPM of stimulated cultures over relevant unstimulated controls.

#### *Supernatant Cytokine, sCD25 and Nuclear Matrix Protein Detection*

Cytokines were assessed from cell culture supernatants in a multiplex format utilizing the Luminex^100^ xMAP System (Austin, TX), with a multiplexed kit (Beadlyte® Human 22-plex Multi-Cytokine Detection System 4; Upstate Biotechnology). Samples were diluted 1∶2 in AIM V medium prior to analysis. sCD25 levels were determined by ELISA according to manufacturer instructions (BD Biosciences). Samples were diluted (when necessary) in PBS containing 10% FBS (pH 7.0). To identify a normal range of sCD25 in human serum, 60 samples were randomly selected from a healthy control population of adolescents and adults, whose origins were as previously described [23]. Soluble nuclear matrix protein 41/7 was measured by ELISA (Calbiochem, San Diego, CA) according to manufacturer instructions.

#### *Reagents*

Recombinant sCD25 was obtained from R&D Systems (Minneapolis, MN) for suppression assays, and from Spring Biosciences for PBMC proliferation assays. Recombinant MMP-2, MMP-9 and the selective MMP2/9 inhibitor were acquired through Calbiochem (San Diego, CA).

### Ethics

Institutional Review Board (IRB) approved informed consents were obtained in accordance with approved protocols.

### Statistical Methods

Statistical analyses utilized GraphPad Prism 4.00 software (GraphPad, San Diego, CA), with Student's paired t tests or ANOVA with the Bonferroni post hoc correction for multiple comparisons along with Spearman's correlation analyses. For all tests, *p*<0.05 was deemed significant.

## Results

### Treg Cells Require Serum for Optimal Suppression of T Cell Proliferation

To investigate sCD25 production during the *in vitro* suppression assay, peripheral blood CD4^+^CD25^−^ conventional T cells (Tconv) and CD4^+^CD25^Hi^ Treg were isolated by FACS. The isolated Treg population demonstrated modestly lower CD4 expression and CD25^Hi^ surface expression, which enriches for cells expressing FOXP3 (Figure S1). To analyze the production of *de novo* sCD25 produced during the suppression assay from the levels already present in healthy control human serum (2039.0±899.8 pg/ml), assays were conducted under serum-free media (SFM) conditions or in SFM supplemented with specified concentrations of autologous serum.

**Figure 1:**
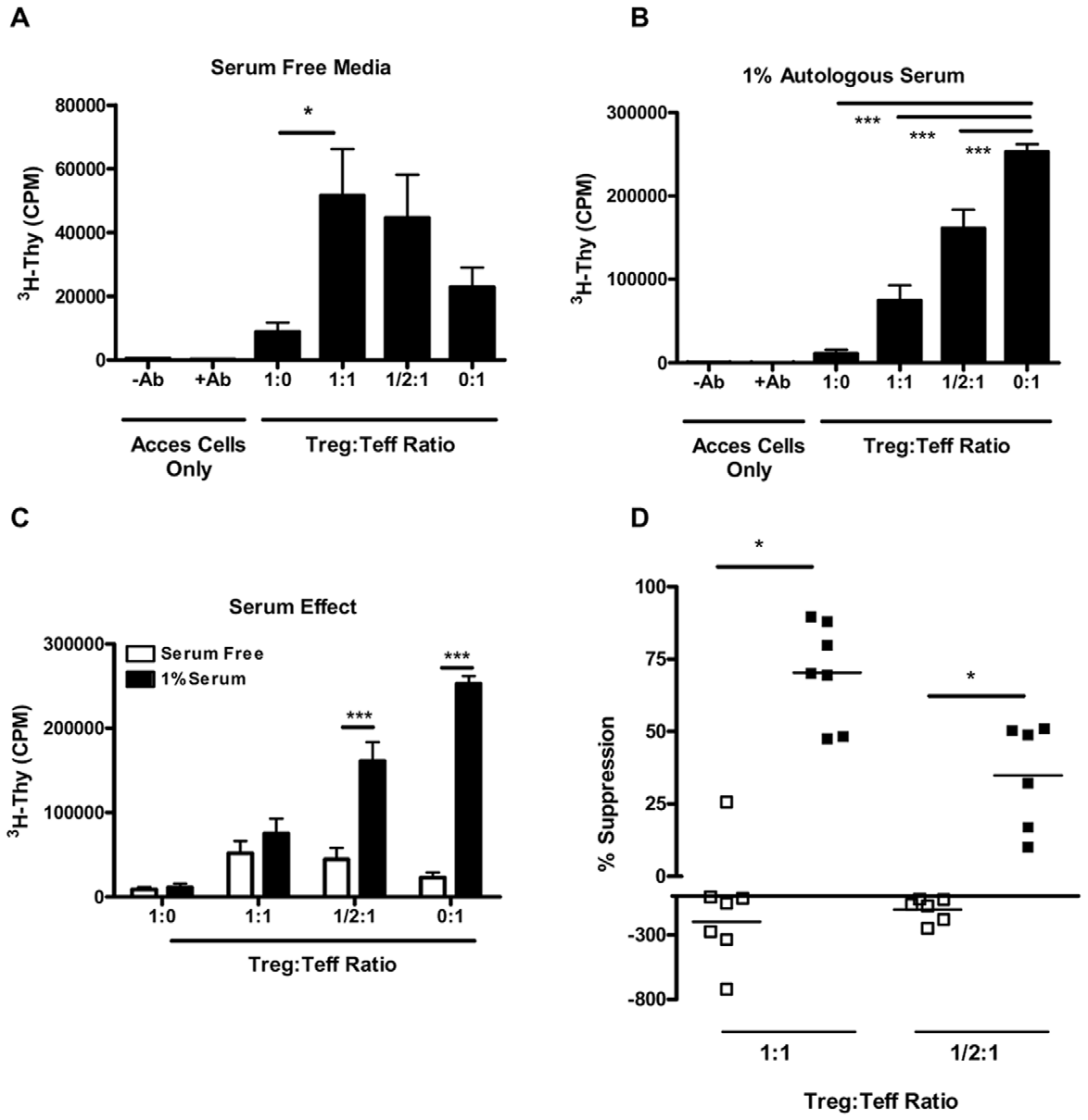
CD4^+^CD25^+^ Treg cells require serum for suppression of T cell proliferation. Suppression assays were conducted utilizing FACS sorted cells run in parallel under either (A) SFM or (B) SFM supplemented with 1.0% autologous serum (n = 7 healthy controls). Treg cells were plated alone (5×10^3^ cells/well), and in decreasing ratios (1∶1, ½∶1, and 0∶1) to a constant number of Teff cells. Cells were stimulated with soluble anti-CD3 (5.0 µg/ml) and anti-CD28 (2.5 µg/ml) in the presence of a ten-fold excess (5×10^4^) of irradiated accessory cells. (C) Graph indicates data plotted from panels (A) and (B) to highlight the differences in proliferation between serum free media (open bars) and 1% serum (closed bars). Cells proliferated significantly less in SFM at Treg to Teff ratios of ½∶1 and 0∶1 (p<0.001 for both conditions). (D). Graph indicates the percent suppression calculated under serum-free conditions (*open squares*) and supplemented with 1.0% serum (*closed squares*) at a ratio of 1∶1-Treg to Teff cells (left data points) and at a ratio of ½∶1 Treg∶Teff cells (right points). Under serum free conditions, Treg fail to suppress proliferation and often lead to increased proliferation in the co-culture (% suppression  =  mean + SEM, −197.8+270.6 for SFM vs. 70.4+17.3 with 1.0% serum; *p = 0.04 at a ratio 1∶1 Treg to Teff. This trend continued at a ratio of ½∶1 Treg to Teff cell (−103.1+91.5 vs. 34.8+18.12, for SFM and 1.0% serum, respectively; **p = 0.01). (*p<0.05, **p<0.01, and ***p<0.001).

Surprisingly, serum absence markedly abrogated the ability of Tregs to suppress proliferation as observed in ^3^H-thymidine-based suppression assays (Figure 1A). This is in contrast to the standard suppressive response; an assay whose outcomes could be fully recovered by adding small (i.e., 1.0%) amounts of serum (Figure 1B). Proliferation was significantly reduced in SFM compared to those containing serum (Figure 1C). The reduced capacity of Treg to suppress under serum free conditions could be a result of altered cellular survival. To examine this possibility, we measured a marker of cellular apoptosis and necrosis during the suppression assay in the presence and absence of serum. Nuclear matrix protein (NMP) levels in culture supernatant were as follows in SFM: ((mean +/− SEM) 1∶0 Treg to Teff = 57.7+/−18.8, 1∶1 = 279.3+/−46, 0∶1 = 254.3+/−35) and in 1% autologous serum: ((mean +/− SEM) 1∶0 = 39.6+/−10.7, 1∶1 = 193.7+/−16.9, 0∶1 = 185.3+/−51.5). Teff cell death did not change in coculture with Treg, suggesting that the altered suppression patterns observed in SFM were unlikely a result of significant changes in cell death. Moreover, Tregs alone did not exhibit significantly increased death in SFM alone. In fact, under SFM conditions, Treg and Teff co-cultures often exhibited increased proliferation over Teff cells alone (Figure 1D; % suppression at a 1∶1 Treg to Teff ratio; mean + SEM, −197.8+270.6 for SFM vs. 70.4+17.3 with 1.0% serum; *p* = 0.04; Student's paired T test). This response was also observed when cells were cultured at a ratio of ½∶1 Treg to Teff cells (−103.1±91.5 vs. 34.8±18.12, for SFM and 1.0% serum, respectively; *p* = 0.01).

The degree of *in vitro* suppression was dependent on both Treg and Teff cell subsets. We noted a dramatically reduced proliferation of Teff cells in SFM versus those in 1.0% serum (Figure 1A and 1B, 0∶1 condition; mean CPM  = 22,840+16,456 for SFM vs. 252,755+24,207 with 1.0% serum, *p* = 0.0006), whereas Treg proliferation was negligible irrespective of the presence of serum when Treg were cultured alone.

### Treg Cells Exhibit Reduced Suppressive Properties and Proliferate when Cultured with Teff Cells under Serum Free Medium Conditions

**Figure 2:**
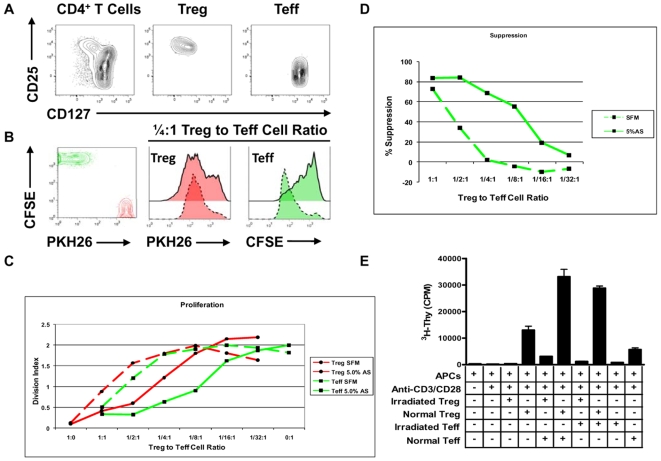
Contribution of Treg and Teff cells to increased proliferation in suppression assay co-cultures. Peripheral blood CD4^+^ T cells ((A), left plot) were FACS sorted based on surface expression of CD25 (y-axis) and CD127 (x-axis) to yield CD4^+^CD127^lo/−^CD25^+^ T cells ((A), middle plot) and CD4^+^CD127^+^CD25^−^ Teff cells ((A), right plot). Following isolation, Teff cells were labeled with CFSE ((B), left plot, y-axis) and Treg cells labeled with PKH26 ((*B*), left plot, x-axis). Proliferation of each gated CD4^+^ T cell population was then assessed following activation conditions utilized in the suppression assay. (*B*) Left plot indicates fluorescence levels of unstimulated cells (depicted in red for Treg and green for Teff), and following 96 h of *in vitro* activation ((B), right plots). Proliferation of Treg and Teff cells are shown at a ¼∶1 Treg to Teff cell ratio and plotted under conditions of SFM (dashed lines) or in the presence of 5% serum (solid lines). (C) For all ratios of Treg to Teff cells, Treg maintain increased suppressive potential and anergic properties in the presence of serum as measured by division index (DI). (D) *In vitro* suppression calculated from DI indicated increased suppression by Treg in the presence of serum. Data plotted represent one example of five independent experiments. (E) A standard suppression assay was set up under SFM conditions with either standard or irradiated (3300 rads) Treg and Teff cells as indicated. This analysis demonstrated a synergistic response at a 1∶1 Treg to Teff ratio, with near maximal proliferation following addition of irradiated Teff cells. Statistical significance indicated as *p<0.05, **p<0.01, and ***p<0.001.

To assess the individual contributions of Treg and Teff to proliferation in co-culture, these cells were FACS sorted (Figure 2A) and then subjected to specific dyes (i.e., the red dye PKH26 for Treg, green dye CFSE for Teff) (Figure 2B, *left plot*). *In vitro* suppression assays were performed and proliferation tracked by the loss of cellular fluorescence in each responding CD4^+^ T cell population (Figure 2B–2D).

In this system, CD4^+^CD127^−/lo^CD25^+^ T cells become anergic in the presence of serum (Figure 2B, 1/4∶1 Treg to Teff cell ratio; dashed green histograms representing culture in SFM and solid overlaid histogram representing culture in 5.0% serum). Proliferation of PKH26 labeled Treg and CFSE labeled Teff cells were tracked according to division index (DI) and plotted for all ratios of Treg to Teff cells during the *in vitro* suppression assay (Figure 2C).

In an effort to more accurately measure the contribution of each cell population to the increased proliferation, we also established a suppression assay in SFM with either normal or irradiated Treg and Teff cell populations (Figure 2E). This analysis indicated that increased proliferation within the CD4^+^CD25^+^ T cell fraction is predominantly responsible for the synergistic response observed in the co-culture as similar proliferation levels were detected in the presence of normal or irradiated Teff cells (33120.0±4866.0 vs. 28828.33±1382.0 CPM, *p* = NS).

### Production of sCD25 During Suppression Assay

**Figure 3:**
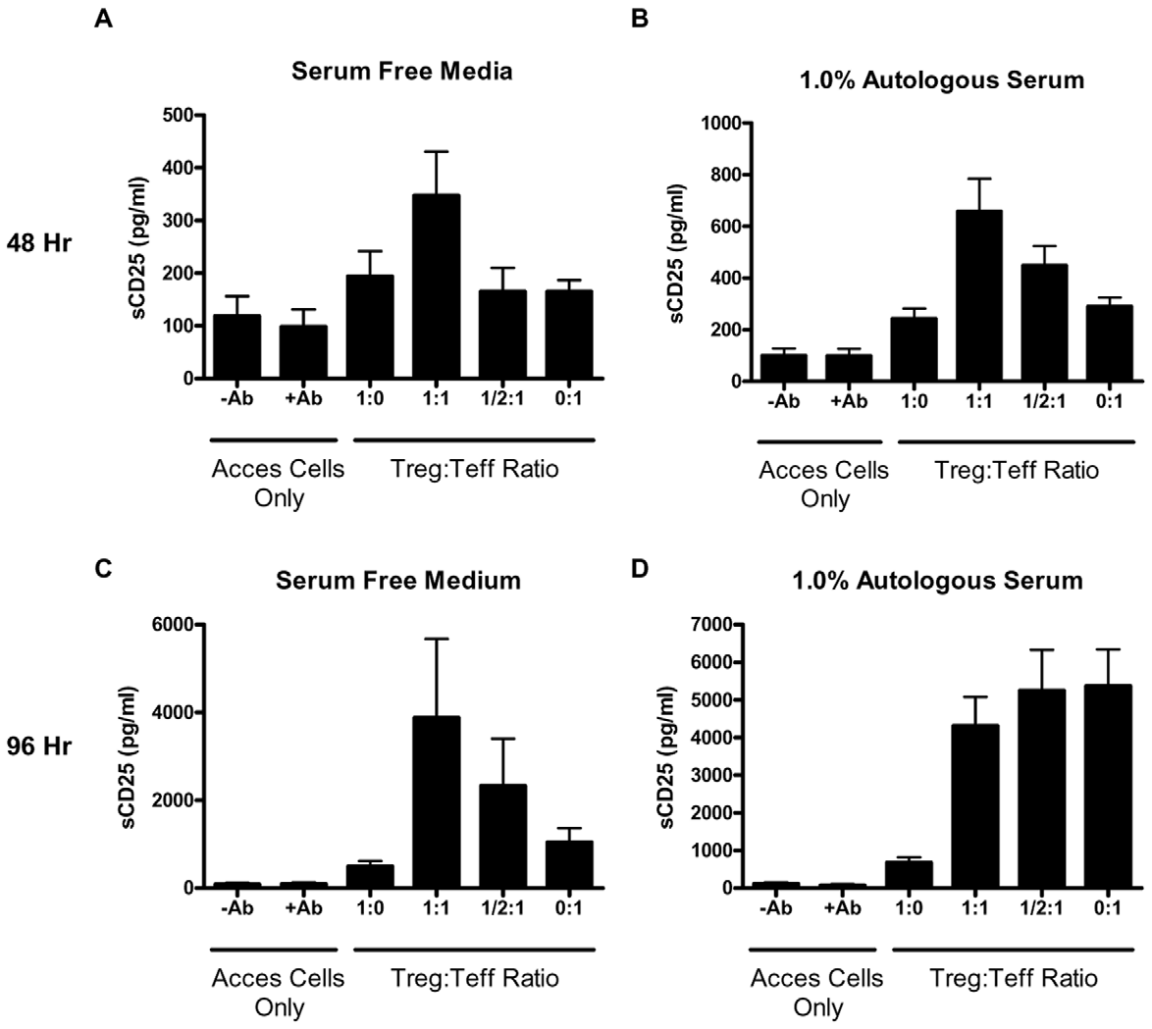
Production of sCD25 during the *in vitro* suppression assay. Supernatants from triplicate wells were pooled and analyzed for the production of sCD25 by ELISA (n = 7 control subjects). Under conditions of both serum-free and 1.0% serum, the highest levels of sCD25 were detected in the 1∶1 Treg to Teff co-culture condition at the early 48 h time point, but were only significantly higher in media containing 1% serum ((A) and (B)). At the 96 h time point ((C) and (D)), the pattern of sCD25 production more closely resemble the responses observed in the proliferation assay assessed by uptake of ^3^H-thymidine. (*p<0.05, **p<0.01, and ***p<0.001).

Treg and conventional T cells have classically been selected based upon their expression of the membrane-bound form of CD25 under baseline conditions [1]. In spite of this, little is known about what happens to soluble and membrane CD25 during the *in vitro* suppression assay. We collected and analyzed supernatants from each condition during the suppression assay in order to determine sCD25 production at 48 and 96 h time points (Figure 3). Overall, this analysis indicated that Treg cells, which were selected based upon high membrane CD25 expression levels, maintain CD25 in a membrane-bound form following polyclonal activation *in vitro* (Figure 3, 1∶0 conditions). Teff cells, on the other hand, are known to upregulate CD25 in response to activation, but then subsequently “shed” it into the tissue culture medium (Figure 3D, 0∶1 condition).

This type of analysis highlights the key influence of culture conditions and timing on the production of sCD25. At an early time point of 48 h, the highest levels of sCD25 are uniformly observed in the 1∶1 Treg to Teff cell co-culture under SFM and serum supplemented conditions (Figure 3A,B). At a later time point of 96 h, the detection of sCD25 more closely resembled the proliferation data observed in the suppression assays (Figure 3C,D). In this assay, the absence of serum resulted in detection of the highest levels of sCD25 in the 1∶1 Treg to Teff co-culture condition (Figure 3C; mean + SEM, 3880.9+4756.5 pg/ml vs. 1043.3+863.8 for Teff cells alone). In line with the proliferation results in the presence of serum, the highest levels of sCD25 were also detected from Teff alone wells (Figure 3D, 0∶1 condition; 5369.5+2575.6 pg/ml). A modest level of suppression in sCD25 levels was observed from the co-culture conditions at a ratio of 1∶1 and 1/2∶1 Treg to Teff cells (4311.1+2029.0 and 5245.3+2666.1, respectively). Finally, in line with the anergic and suppressive properties of Treg, the lowest levels of sCD25 were detected from Treg cultures alone (Figure 3D, 682.2+367.1 pg/ml).

### The Production of sCD25 Correlates with Cellular Proliferation in the Suppression Assay

**Figure 4:**
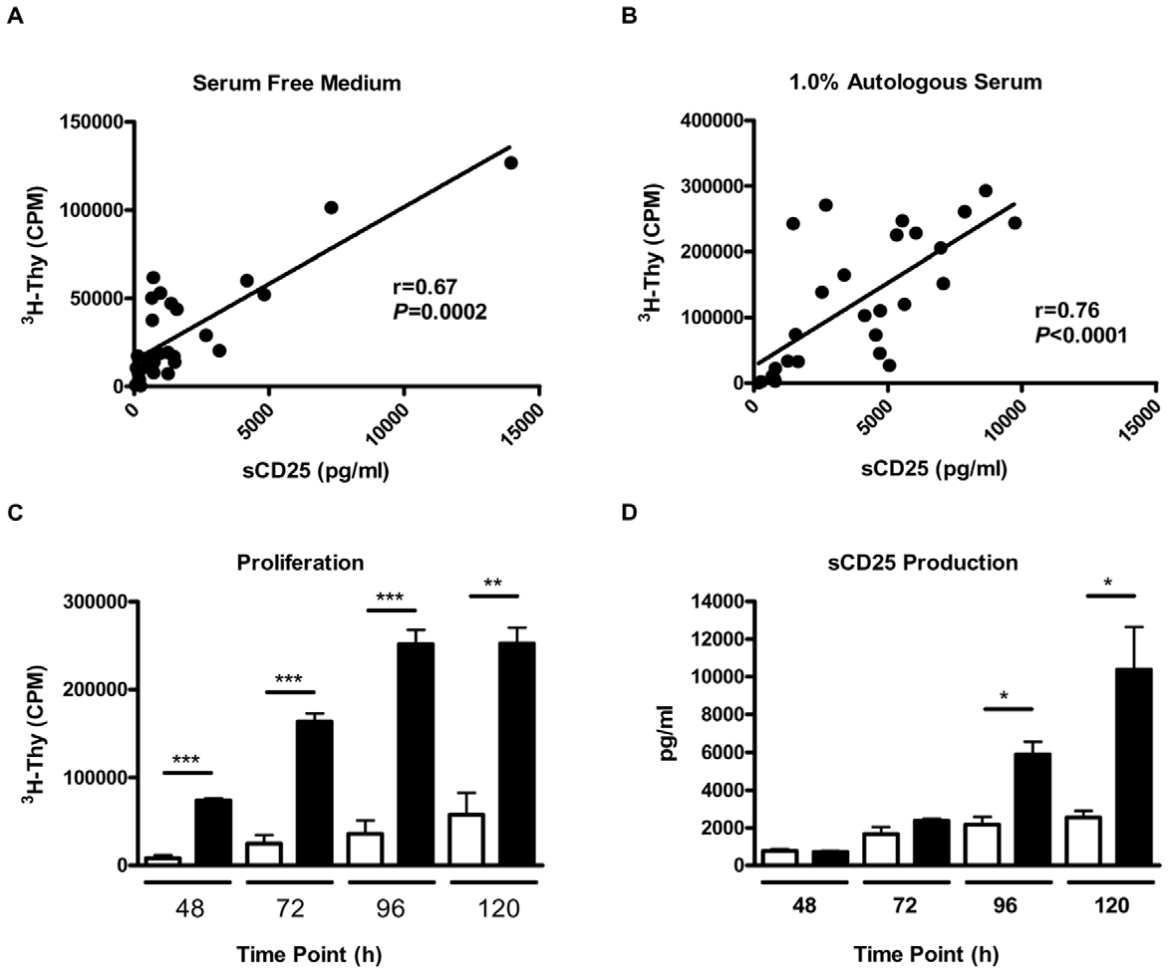
Levels of cellular proliferation correlate with the production of sCD25. Levels of sCD25 produced during the *in vitro* suppression assay were plotted versus the amount of proliferation detected at the 96 h time point. Under both (A) SFM and (B) 1.0% serum conditions, for all ratios of Treg to Teff cells (1∶0, 1∶1, ½∶1, and 0∶1, *n* = 7), the levels of sCD25 detected (x-axis) correlate with cellular proliferation as assessed by the incorporation of ^3^H-Thymidine (y-axis). Isolated Treg and Teff cells were then assessed for their capacity to proliferate and produce sCD25 in an autocrine fashion over a 5 day time period. Purified CD4^+^CD25^+^ (open bars) and CD4^+^CD25^−^ T cells (closed bars) were cultured under expansion conditions utilizing beads coated with anti-CD3 and anti-CD28 as well as exogenous human recombinant IL-2 (300 U/ml) under serum-free conditions. Shown are (C) proliferation and (D) sCD25 production at 48, 72, 96, and 120 h time points by each indicated cell population. The data plotted represent the mean CPM and pooled sCD25 levels of replicate cultures (*n* = 6 individuals) with situations identifying significance between Treg and Teff cells indicated (*p<0.05, **p<0.01, and ***p<0.001).

Considering the similar patterns of cellular proliferation and sCD25 production during the *in vitro* suppression assay, we sought to determine the relationship between proliferation and sCD25 production. Under both serum-free and serum-supplemented conditions, the production of sCD25 at 96 hours correlated with the levels of cellular proliferation observed under all ratios of Treg to Teff cells (Figure 4A and 4B; r = 0.67, *p* = 0.0002 for SFM and r = 0.76, *p*<0.0001 for 1.0% serum).

In order to determine the cellular source of sCD25, freshly isolated CD4^+^CD25^−^ and CD4^+^CD25^+^ T cells were stimulated individually in the presence of anti-CD3 and anti-CD28 coated beads and exogenous IL-2. This analysis revealed that sCD25 can be generated in an autocrine fashion from both Treg and Teff cells following activation in the absence of any other cell types (Figure 4). We found it intriguing that the culture conditions which abrogate the anergic and suppressive properties of Treg (i.e., by provision of cross-linking anti-CD3 and anti-CD28 co-stimulation, as well as exogenous IL-2) represent the same conditions that elicit the production of sCD25 from purified Treg. It should once again be noted, however, that both proliferation and sCD25 production were lower in Treg cells during expansion cultures when compared to Teff cells (Figure 4C and 4D). This is particularly apparent at the later time points of 96 and 120 h, when Treg likely have consumed exogenous IL-2 (Figure 4D). We would also note that production of sCD25 appears specific for the growth factor present (herein IL-2), as production of sCD127 (the IL-7R alpha-chain) was detected near background levels following robust *in vitro* expansion (data not shown).

**Figure 5:**
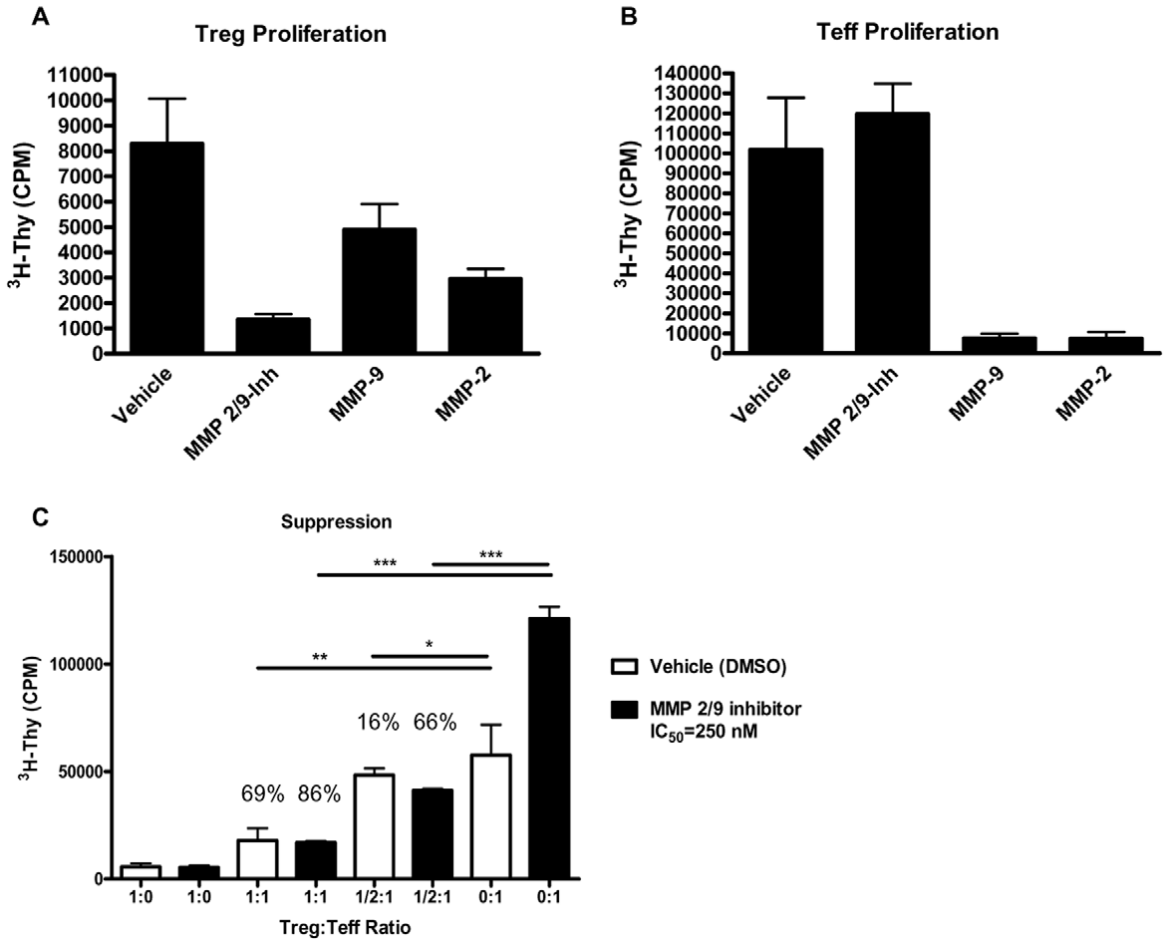
Alteration of MMP-2 and MMP-9 activity alters Treg and Teff cell proliferation and *in vitro* suppression. FACS isolated Treg (A) and Teff (B) were activated with anti-CD3 and anti-CD28 coated microbeads in the presence of vehicle control or the selective MMP-2/9 chemical inhibitor II (IC_50_ = 250 nM). Addition of active recombinant MMP-2 and MMP-9 (1 µg/ml each) led to reduced proliferation of Treg (A) and significantly reduced proliferation of Teff (B) cells. The inhibitor reduced proliferation of Tregs (p<0.01) while having no effect on Teffs. (C) The addition of MMP-2/9 inhibitor to the *in vitro* suppression assay resulted in increased Teff proliferation while at the same time, augmenting Treg-mediated suppression. Percent (%) suppression at 1∶1 (Treg/Teff) and ½∶1 ratios are noted above the bars, as a comparison to vehicle (0∶1) or MMP-2/9 inhibitor (0∶1) CPM. Graphs show one representative experiment of three individual experiments. (*p<0.05, **p<0.01, and ***p<0.001).

Finally, given a previous report suggesting MMP-2 and MMP-9 could elicit CD25 cleavage [15], we analyzed the capacity of these two proteases to modify Treg and Teff cell responses. Consistent with that report, we noted that addition of MMP-2 or MMP-9 *in vitro* reduced Treg (Figure 5A, *p*<0.05 vs. vehicle) and to an even greater degree, Teff cell proliferation under anti-CD3/anti-CD28 stimulation (Figure 5B, *p*<0.05 vs. vehicle). Interestingly, addition of a selective MMP-2/9 chemical inhibitor trended towards an increase in Teff proliferation (Figure 5B, albeit *p* = NS), whereas Treg proliferation was significantly reduced by addition of this inhibitor (Figure 5A, *p*<0.01). Consistent with this notion, addition of the MMP-2/9 inhibitor to the standard *in vitro* suppression assay increased the proliferative capacity of Teff, while augmenting the suppressive function of Treg (Figure 5C).

### Treg Maintain the Capacity to Suppress Teff Cell Cytokine Production in Serum Free Media Conditions

In addition to suppressing proliferation, one of the hallmark phenotypes of Treg is their capacity to suppress cytokine production by Teff cells [25]. In light of the findings of deficient suppression of proliferation by Treg in SFM conditions, we questioned whether Treg were also deficient in their capacity to suppress cytokine production by Teff cells. To test this notion, supernatants were collected and analyzed for the production of a panel of 22 cytokines by multiplex analysis (complete cytokine results are contained in Table S1).

Surprisingly, Treg maintained their capacity to suppress Teff cell cytokine production under both SFM and serum supplemented conditions for a majority of cytokines analyzed. However, some cytokines did exhibit increased production in co-culture under SFM conditions, paralleling the proliferation results. In this case, it is of interest to note that these responses tended to include cytokines classically associated with a T_R_1 or T_H_2-associated phenotype (e.g., IL-10 and IL-5). It is also worth noting that despite the highest levels of proliferation in the Treg and Teff co-culture condition (1∶1 in SFM), the highest levels of IL-2 were still detected from Teff cells alone (mean ± SEM, 11.7±10.2 pg/ml for the 1∶1 Treg to Teff cell condition vs. 129.6+155.5 for Teff only). On the other hand, despite dramatically higher Teff cell proliferation in the presence of serum (Figure 1C, 0∶1 conditions), little detectable IL-2 was observed at the 96 h time point in supernatants.

### Exogenous sCD25 Does Not Affect Suppression, but Alters Proliferation of Teff Cells

**Figure 6:**
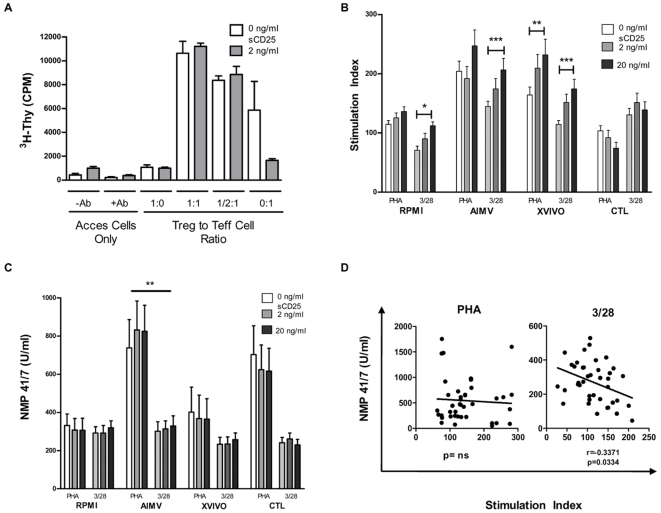
Addition of soluble CD25 differentially effects proliferation of Teff cells and PBMC. A suppression assay was set up under serum-free media ((A), open bars), or in the presence of a human recombinant sCD25 protein ((A), closed bars). Addition of sCD25 did not significantly restore Teff cell responses or the anergic properties of Treg in serum-free conditions (*p* = NS for all Treg to Teff cell ratios, 1∶0, 1∶1, ½∶1, and 0∶1). In the 0∶1 condition, sCD25 did appear to reduce proliferation. sCD25 was added to PBMC cultures (n = 10) in the presence of increasing concentrations of sCD25, with anti-CD3/28 microbeads ((B), open bars) or PHA ((B), hatched bars) stimulation. Proliferation increased in some culture conditions and decreased in others. Statistically significant increases were seen by ANOVA under 3/28 stimulation in RPMI (*p* = 0.05), AIMV (*p* = 0.001), and XVIVO (*p* = 0.001) media, and under PHA stimulation in XVIVO medium (*p* = 0.01) when comparing 0 and 20 ng of sCD25 added. Comparisons among the same stimulation showed that cells cultured in RPMI and AIMV media had significant differences in proliferation regardless of the amount of sCD25 added, with both PHA and CD3/28 stimulation. Under PHA stimulation, CTL cultures proliferated less than either XVIVO or AIMV cultures. To determine if this differential proliferation was due to altered cell death, we measured the amount of nuclear matrix proteins (NMPs) released into the supernatant by dying cells (C). Addition of sCD25 did not result in a dose-responsive increase in cell death. The outcome varied depending on the culture media and stimulus. Plotting the stimulation index versus the production of NMP showed a negative correlation between proliferation and cell death under CD3/28 stimulation ((D), bottom panel). *p<0.05, **p<0.01, and ***p<0.001.

We sought to determine the effect of sCD25 supplemented at “serum” concentrations on Treg function in the suppression assay (Figure 6A). The addition of sCD25 did not affect suppression, but did result in decreased proliferation in Teff cells alone (0 ng/ml = 5865±2410 CPM vs. 2 ng/ml = 1656±143.9, *p* = NS). These results conflicted with what has been reported in the literature [12].

### Exogenous sCD25 has Variable Effects on PBMC Proliferation and is Dependent on the Culture Milieu

Our previous data reflecting the influence of serum presence along with the trend seen in (Figure 6A) led us to investigate the effects of multiple culture conditions and stimuli on production and function of sCD25. To reflect native immune conditions rather than isolated effects, we used total PBMC (n = 10) and direct TCR stimulus (anti-CD3 and anti-CD28 coated beads) as well as a mitogenic T cell stimulus (PHA-L). We chose four media formulations to compare the results between these assays - standard conditions with RPMI 1640 with 5% human AB serum, as well as three different serum free media - AIMV, XVIVO, and CTL Test media. sCD25 was added at 0, 2, and 20 ng/ml to approximate the levels present in serum and those that may persist at areas of inflammation. With direct TCR stimulus (Figure 6B, hatched bars), the high dose of sCD25 significantly increased proliferation as compared to the control by ANOVA in RPMI (0 ng/ml = 70.85±6.894 vs. 20 ng/ml = 112.2±6.55, *p*<0.05), AIMV (0 ng/ml = 144.7±8.695 vs. 20 ng/ml = 206.6±19.33, *p*<0.001) and XVIVO media (0 ng/ml = 114.4±6.453 vs. 20 ng/ml = 174.4±16.33, *p*<0.01). Under mitogenic stimulus (Fig. 6B, open bars), only cultures in XVIVO medium exhibited increased proliferation (0 ng/ml = 164.1±13.39 vs. 20 ng/ml = 231.8±26.55, *p*<0.001) while showing a decreasing trend in CTL serum free medium (Figure 6B). These comparisons indicate that substrate as well as matrix constituents in media lead to significantly different proliferation.

### Exogenous sCD25 May Increase Survival in PBMC Cultures

To determine if this proliferation difference was due to differential cell death in these cultures, nuclear matrix protein 41/7 (NMP 41/7) was measured in supernatant by ELISA (Figure 6C). Increasing concentrations of sCD25 alone had no affect on cell death by ANOVA (*p* = NS for all comparisons). Comparisons following stimulation in the various culture medium revealed that cell death could be altered by both factors: stimulus (AIMV: 0 ng/ml = 738.1±47.9 (with PHA) vs. 301.1±49.33 (3/28), *p*<0.01; 2 ng/ml = 832.2±151.8 (PHA) vs. 313.6±42.36 (3/28), *p*<0.01; 20 ng/ml = 824.9±136.7 (PHA) vs. 329±53.41 (3/28); ANOVA) and media formulation only with mitogenic stimulus (PHA) (AIMV = 798.4±30.24 vs. RPMI = 315.2±8.172 and vs. XVIVO = 378.2±11.64, both *p*<0.05; ANOVA). The stimulation index correlated with cell death only with CD3/28 stimulation (Figure 6D, r = −0.3371, *p* = 0.0334). Interestingly, in comparisons between media formulations, there were both positive and negative correlations that were statistically significant. Cells cultured in AIMV and XVIVO media showed decreased proliferation with increased cell death, whereas PHA stimulation in CTL medium resulted in increased proliferation with increased death (data not shown). This suggests that sCD25 at high concentrations in local microenvironments may protect T cells from AICD, thus allowing increased viability and proliferation.

### Production of sCD25 by PBMC Correlates with Cell Death

**Figure 7:**
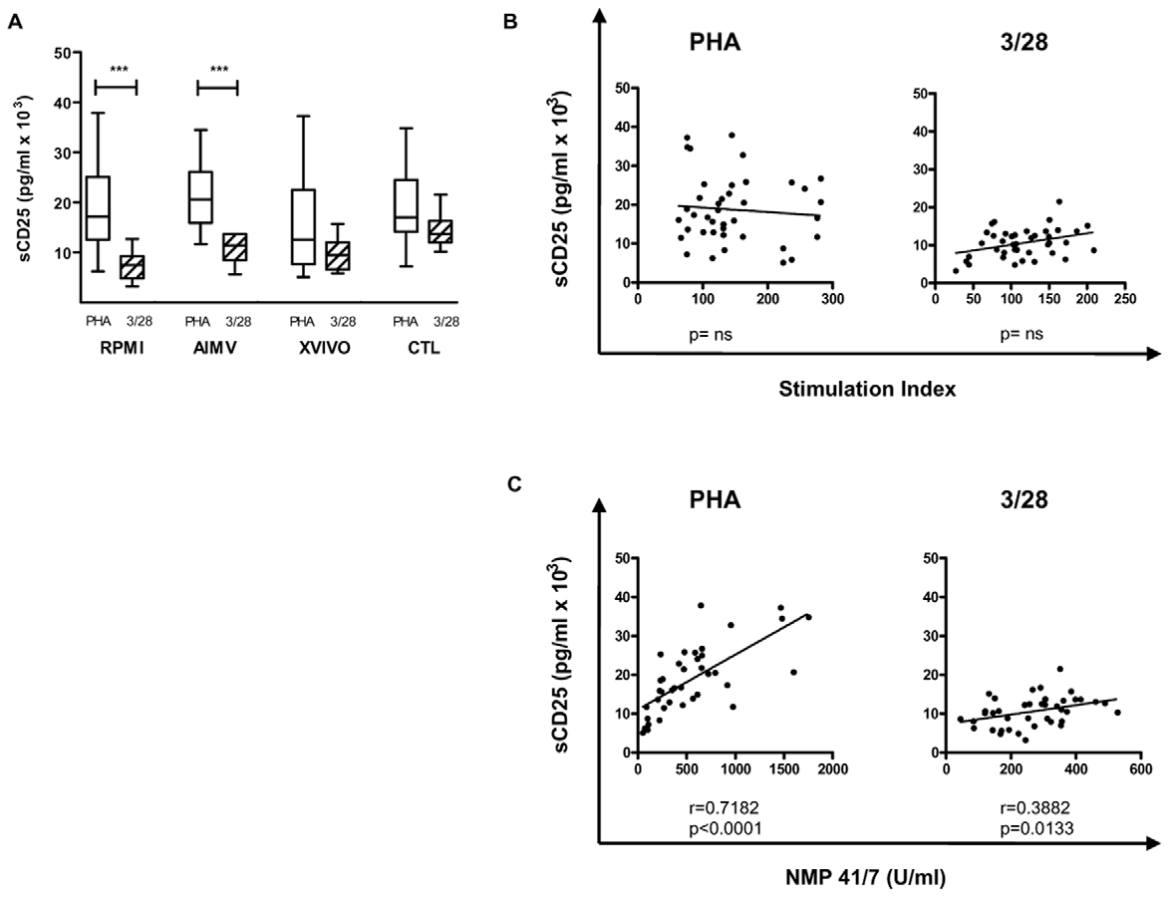
sCD25 production in the absence of exogenous recombinant protein correlates with cell death, not proliferation, of PBMC. To determine if the variable results seen previously were present in other areas, we measured the production of sCD25 in the 0 ng/ml condition with anti-CD3/28 microbeads ((A), open bars) or PHA ((A), hatched bars) stimulation. sCD25 production varied dependent on the stimulus provided in RPMI and AIMV media conditions (RPMI: (PHA) 18952±2872, (3/28) 7457±916.8, p<0.001; AIMV: 21482±2114, (3/28) 10957±893.8, p<0.001), but did not significantly differ between media formulations. In (B), sCD25 production did not correlate with proliferation as seen previously. Rather, sCD25 was generated at levels correlating with cell death as measured by NMP production, (C) (PHA: r = 0.7182, *p*<0.001, CD3/28: r = 0.3882, *p* = 0.0133). ***p<0.001.

The differing proliferation results in PBMC cultures as compared to isolated T cell cultures led us to investigate the production of sCD25 as a function of PBMC proliferation (Figure 7). sCD25 levels measured in cultures with no exogenous sCD25 (Figure 7A) varied in response to different stimuli in both RPMI and AIMV medium (RPMI: PHA = 18952±2872 vs. 3/28 = 7457±916.8, p<0.001; AIMV: PHA = 21482±2114 vs. 3/28 = 10957±893.8, *p*<0.001). Comparison to proliferation showed that the correlation seen earlier was absent in PBMC (Figure 7B, *p* = NS by linear regression and Spearman correlation). Instead, production of sCD25 correlated with cell death (Figure 7C, PHA: r = 0.7182, *p*<0.001, CD3/28: r = 0.3882, *p* = 0.0133).

## Discussion

In this report, we describe a critical facet influencing T lymphocyte function; that being the differential control of membrane and soluble forms of CD25 on T cell subsets. We observed that CD25, which has commonly been used to select functional Treg, remains stable on the surface of anergic and suppressive Treg during the *in vitro* suppression assay. On the other hand, CD25 is upregulated on recently activated Teff cells, but is then subsequently released into the culture medium. These findings imply that Treg may obtain quantitatively and qualitatively greater levels of IL-2 signaling compared to Teff cells as a result of increased membrane CD25 stability and affinity for IL-2. These data also suggest that IL-2 production by Teff cells is required to drive continued cell cycle progression and sCD25 production by both cell populations.

In an effort to isolate the production of sCD25 from the levels added through serum supplementation, we conducted these experiments in a complete SFM formulation. To our surprise, the absence of serum impaired the in *vitro* suppression of proliferation assessed by standard ^3^H-thymidine-based assays but not Teff cell cytokine production. In terms of developing an explanation for this phenomenon, we proposed three non-mutually exclusive explanations. First, Tregs may be more sensitive to a lack of serum components during co-culture. Second, Tregs may be contaminated with non-regulatory T cell populations that are relatively serum independent. Third, Tregs lose their anergic and suppressive properties under serum free conditions. In testing these three potential explanations we noted that the degree of *in vitro* suppression simultaneously takes into account the responses of Treg and Teff cells. Here, we observed functional deficiencies in both populations under SFM conditions, with a marked hyporesponsiveness of Teff cells and a concomitant failure by Treg to remain anergic and suppress proliferation. Despite deficient responses of both populations during in vitro suppression, cell tracking dye studies and markers of apoptosis/necrosis did not indicate significant differences in cell death due to the absence of serum. The addition of increased sorting stringency by inclusion of CD127 did restore some of the suppressive capacity of Tregs under serum free conditions, implying some contribution by non-Tregs when only CD4 and CD25 were used to sort. However, cell dilution dye assays tracking proliferation of Tregs and Teff cells during suppression indicated increased cell cycling from the entire population of Tregs in SFM making outgrowth of a small subset during the culture an unlikely explanation for the increased proliferation observed. In sum, the in vitro suppression assay is highly sensitive to potential artifacts due to media components when assessing proliferation alone and may provide only limited insight into the potential in vivo function of Tregs.

In terms of which component(s) within serum are required for full T cell activation as well as suppression by Treg, several candidates are the focus of our ongoing investigations. One such candidate, TGF-β, serves a wide variety of pleiotropic functions, with a role for TGF-β in immune regulation forming the subject of much recent debate. TGF-β is a major cytokine product of Treg and has also been reported to exist in an active form on the surface of these cells [26]. Furthermore, TGF-β can elicit the conversion of CD4^+^CD25^−^ T cells into FOXP3^+^ T cells [27]. The function of Treg has been reported to be influenced by both the presence and activation state of TGF-β (i.e., in serum, TGF-β often exists in an inactive latent form (TGF-LAP)). In a related concept, the balance of serum proteases and protease inhibitors represent additional components which may serve dual roles by influencing the cleavage of CD25, as well as through their capacity to modify the activation state of TGF-β [15], [28]. To the existing proteases reported to cleave CD25 [15], [16], here we add our findings that addition of MMP-9 or MMP-2 has effects on both Treg and Tconv cell responses. In addition, our data suggest that chemical inhibition of specific proteases may also influence CD25 stability and immune responsiveness. Controlling the proteolytic release of CD25 from the surface of Treg or Teff cells may provide novel therapeutic targets to alter IL-2 receptor signaling.

These findings raise the intriguing question of why CD25 appears to be more stable on the surface of Treg compared to Teff cells following stimulation and how this observation influences the downstream function of these cells. The IL-2 signaling axis can elicit proliferation of T cells, but also contains at least two mechanisms for controlling T cell expansion in response to antigen. IL-2/CD25 interactions reinforce the metabolic fitness of Treg, leading to dominant immune suppression [29]. In Teff cells, excess IL-2 signaling can lead to AICD. We would speculate that the CD25 shedding from freshly activated Teff cells observed in these studies may be necessary to prevent these processes, allowing for continued cycling as well as maintaining activation requirements in daughter cell populations.

If a stable IL-2 signal reinforces FOXP3 expression and Treg function, one might expect situations that interrupt IL-2 signaling to interfere with Treg activity. An example of this situation was reported by Tang and colleagues who observed that intra-islet Treg cells expressed reduced amounts of CD25 and Bcl-2, which led to a functional imbalance of Treg and Teff cell populations leading to the development of type 1 diabetes in non-obese diabetic mice [30]. In addition, multiple reports in inflammatory autoimmune diseases and studies of human T-cell leukemia virus type-I (HTLV-1) associated lymphoma report elevated serum levels of sCD25 and defective suppression by Treg [31]–[33]. Therefore, it appears consistent through multiple disease processes that disturbances in the IL-2 and CD25 signaling axis are often paralleled by defective immunoregulation by Treg. Some of the factors which may influence CD25 stability, and subsequent immune reactivity, are modeled in Figure S2.

As mentioned previously, at least two major hypotheses on the formation of sCD25 exist. Either Treg express a membrane-stable form of CD25, or Treg cultures fail to elicit the factors that lead to the proteolytic cleavage of CD25. Tregs are capable of producing sCD25 upon activation and IL-2 exposure suggesting that production of IL-2 by Teff cells may account for their continued sCD25 production and proliferation. These observations taken during the *in vitro* suppression assay appear to indicate that not only are adaptive cytokine levels important for T cell proliferation and function but in addition, the stability of cytokine receptors warrants further consideration.

The influence of serum seen in suppression assays indicated that a more thorough investigation of culture conditions was required to accurately compare inter-laboratory data. Therefore, PBMC responses were measured under TCR or mitogenic stimulation under various culture conditions. What we have described is that the presence or absence of serum as well as the components of the culture media influenced the data collected in terms of sCD25 production, proliferation and cell death. Overall, the addition of exogenous sCD25 increased proliferation in three out of four culture conditions. The results of these assays are difficult to interpret, in that slight modifications in the *in* vitro environment have an effect on multiple outcome measures. This may provide an explanation for the variation between laboratory results, but likely does not complete the story. Indeed, the presence of sCD25 appears to have differential effects on pre-activated naive T cells versus whole PBMC.

These studies highlight the importance of the soluble and membrane associated forms of CD25 in terms of influencing cell proliferation, survival, and suppression via Treg. The extensive variability between investigations as well as various culture conditions highlights the need for increased standardization during *in vitro* assays. This is also contingent upon a more complete understanding of the *in vivo* microenvironments controlling immune responses. In terms of future implications, these studies highlight important facets to consider in diseases associated with abnormal levels of sCD25 as well as defective regulation by Treg.

### Limitations

The role of sCD25 remains obscure because of the variations in culture conditions, and ultimately will have to be resolved *in vivo* by knock-in of only the soluble form into mice. The observation that sCD25 may promote survival is an intriguing possibility, one that might have a basis in the stoichiometric competition for other receptor subunits in the common gamma chain cytokine family, where cleaved CD25 allows for a more homeostatic cytokine (i.e., IL-7, IL-15) signaling. We would argue that the relatively abundant amount of sCD25 in serum would not simply be waste and this is the subject of ongoing investigation.

What we can infer from these experiments though is that sCD25 production on the whole is more likely associated with activation induced cell death than proliferation per se. In light of this, the multiple proteases linked with apoptotic cell death may be involved in releasing CD25 from the cell surface. Chief among these candidates are caspases, which we assume would have access to CD25 upon internalization of the IL-2 receptor. This is particularly pertinent considering recent studies linking disease-associated polymorphisms in *CD25* with increased susceptibility to type 1 diabetes and multiple sclerosis [9], [11]. In the case of type 1 diabetes, we reported susceptible alleles in type 1 diabetes were linked with lower serum levels of sCD25 [9]. In addition, in the non-obese mouse model of type 1 diabetes, allelic variation of *IL-2* was associated with reduced IL-2 expression correlating with impaired Treg activity [34]. Thus, defects within the IL-2/CD25 signaling axis may constitute a conserved immune/AICD defect predisposing to autoimmunity.

This study also draws attention to the necessity for standardization of *in vitro* assays to allow for proper interpretation and comparison of results. Proprietary media formulations in combination with batch and vendor variation in serum supplements hamper *in vitro* comparisons. A recent publication regarding difficulties in *in vitro* polarization of Th17 cells due to media composition shows a problem that is understood but not fully explored – that immune cell activity *in vivo* is highly dependent on the location [35]. Thus, it may be necessary to define *in vitro* conditions that are more truly reflective of the many environments where immune cells perform their functions.

## Supporting Information

Table S1 (0.07 MB PDF)

Figure S1 Isolation of Treg and T conventional cells from human peripheral blood by FACS. Representative plots showing (A) pre-sort expression profiles of CD4 (x-axis) and CD25 (y-axis, left plots) or CD25 (y-axis) and CD127 (x-axis, middle plots) following CD4 negative selection. Plots represent post-sort purity of sorted cells indicating expression of CD4, CD25, CD127, and FOXP3 for (B) T conventional cells and (C) Tregs. Post-sort FOXP3 analysis was assessed immediately following FACS isolation (right plots, black overlays represent isotype control and blue overlays indicate FOXP3 staining, respectively).
(0.77 MB TIF)

Figure S2 Membrane CD25 stability influences immune responsiveness. A hypothetical model is shown depicting how alterations in membrane-bound and soluble forms of CD25 (sCD25) may influence the activities of Treg and conventional T (Tconv) cell subsets. The immunological microenvironment provides costimulatory and cytokine signals through APC which influence CD25 stability on Treg and Tconv cells. Both intrinsic signals, through TCR signal strength and costimulation, as well as extrinsic inflammatory signals such as TOLL ligand induced proinflammatory cytokines may skew T cell phenotypes through the production of proteases and subsequent cleavage of membrane-bound CD25. Loss of membrane CD25 and IL-2 signaling in Treg would result in reduced phosphorylation of STAT5 and downstream FOXP3 expression. Conversely, in Tconv cell populations IL-2 signal strength along with costimulatory and cytokine differentiation signals would influence the lineage fate of responding T cell populations. Loss of CD25/IL-2 high affinity signaling from Teff cell populations would result in lower IL-2 affinity and/or dependence on signaling from other common γ-chain receptors for survival (such as IL-7, IL-15, and IL-21). Conversely, Tconv cells activated under tolerogenic conditions would lead to increased CD25 membrane stability and subsequent FOXP3 expression attenuating effector responses (Th17, Th1, and Th2) and reinforcing an induced Treg (iTreg) phenotype. The signal strength as measured by both affinity and avidity for IL-2 (related to the alpha beta gamma combinations) is important in driving proliferation but also AICD.
(0.53 MB TIF)
