# Supplementary material for: Influence of Membrane CD25 Stability on T Lymphocyte Activity: Implications for Immunoregulation
Source: PLoS One. 2009 Nov 24;4(11):e7980. doi: 10.1371/journal.pone.0007980 (PMC2775921; doi:10.1371/journal.pone.0007980)
Supplement: Table S1 — (0.07 MB PDF) [file pone.0007980.s001.pdf]

Supporting Table 1. Suppression assay cytokine levels

Cytokine		Treg	1:1 Treg:Teff	Teff	% Suppression
Eotaxin	SFM	25.7±15.7*	32.7 ± 30.9	31.9 ± 27.7**	-2.5
	1%AS	22.5 ± 14.4	23.2 ± 13.2	43.4 ± 33.0	46.5
GM-CSF	SFM	6.6 ± 8.6	106.5 ± 70.4	194.2 ± 259.1	45.2
	1%AS	6.5 ± 9.1	38.0 ± 31.8	275.5 ± 118.0	86.2
IFN-γ	SFM	13.7 ± 24.0	1254.6 ± 1578.5	5146.3 ± 5292.8	75.6
	1%AS	20.1 ± 42.3	309.2 ± 267.8	4971.8 ± 2932.7	93.8
IL-10	SFM	10.3 ± 14.2	45.2 ± 63.7	14.0 ± 21.9*	-222.9
	1%AS	20.4 ± 26.8	56.3 ± 57.9	100.5 ± 108.8	44.0
IL-12p40	SFM	61.8 ± 46.4	293.1 ± 322.8	644.4 ± 667.3	54.5
	1%AS	38.8 ± 42.4	146.3 ± 42.3	421.4 ± 161.5	65.3
IL-12p70	SFM	3.8 ± 3.6	4.4 ± 4.0	6.7 ± 7.3	34.3
	1%AS	4.1 ± 2.8	5.1 ± 4.3	6.8 ± 4.4	25.0
IL-13	SFM	42.4 ± 63.4	268.6 ± 273.5	306.1 ± 307.7	12.3
	1%AS	37.2 ± 80.1	123.0 ± 165.7	196.2 ± 156.3	37.3
IL-15	SFM	ND†	ND	ND	
	1%AS	ND	ND	ND	
IL-1α	SFM	26.7 ± 48.9	211.3 ± 253.1	289.6 ± 346.8	27.0
	1%AS	31.9 ± 31.9	146.3 ± 75.3	415.1 ± 94.4	64.8
IL-1β	SFM	5.2 ± 0.1	6.6 ± 2.0	7.9 ± 2.7	16.5
	1%AS	5.0 ± 0.3	8.0 ± 1.7	14.3 ± 10.0	44.1
IL-2	SFM	4.0 ± 5.9	11.7 ± 10.2*	129.6 ± 155.5	91.0
	1%AS	2.4 ± 4.2	2.1 ± 3.6	4.4 ± 6.3	52.3
IL-3	SFM	21.3 ± 20.0	130.8 ± 121.2	258.8 ± 225.2	49.5
	1%AS	11.3 ± 23.4	54.4 ± 45.7	240.3 ± 126.8	77.4
IL-4	SFM	8.8 ± 15.3	11.8 ± 17.2	12.0 ± 18.6	1.7
	1%AS	9.2 ± 16.7	9.2 ± 16.3	9.7 ± 16.5	5.2
IL-5	SFM	19.5 ± 48.0	87.8 ± 110.6	34.7 ± 51.7	-153.0
	1%AS	29.1 ± 71.2	81.1 ± 124.0	86.0 ± 93.0	5.7
IL-6	SFM	13.0 ± 12.9	28.0 ± 25.1	53.5 ± 58.9	47.7
	1%AS	20.2 ± 27.0	29.5 ± 40.0	74.2 ± 83.6	60.2
IL-7	SFM	236.3 ± 50.9	285.0 ± 64.9	270.6 ± 61.2	-5.3
	1%AS	203.4 ± 98.2	275.8 ± 53.8	301.0 ± 29.2	8.4
IL-8	SFM	2251.6 ± 750.6	6363.5 ± 5016.3	6437.0 ± 3993.0	1.10
	1%AS	2187.9 ± 1534.3	10526.8 ± 11170.1‡	5863.3 ± 1406.8	-79.5
IP-10	SFM	390.7 ± 676.1	6882.1 ± 10286.8‡	7693.2 ± 11335.0‡	10.5
	1%AS	345.8 ± 273.5	2165.4 ± 1276.6	9645.5 ± 5837.7	77.6
MCP-1	SFM	157.0 ± 195.6	304.5 ± 255.4*	408.3 ± 278.7**	25.4
	1%AS	221.8 ± 239.9	574.6 ± 431.6	1038.9 ± 551.8	44.7
MIP-1α	SFM	14.2 ± 14.1	371.0 ± 581.2*	1164.8 ± 1937.5*	68.1
	1%AS	23.7 ± 25.4	611.9 ± 754.0	9905.3 ± 7667.8	93.8
RANTES	SFM	76.6 ± 144.0	380.3 ± 655.9	384.4 ± 519.6	1.1
	1%AS	83.0 ± 184.0	291.3 ± 335.4	1082.2 ± 909.7	73.1
TNFα	SFM	1.1 ± 2.6	62.9 ± 102.2	199.1 ± 259.3*	68.4
	1%AS	1.8 ± 3.3	41.2 ± 39.1	502.6 ± 294.8	91.8

Cytokine data represents the mean concentration (pg/ml) + SD (N=7 control subjects). Cytokine levels were determined from culture supernatant pooled from triplicate wells (stored at -20˚C) and subsequently diluted 1:2 prior to analysis. †ND, indicates no detection. ‡ Designates samples with values exceeded the top standard for the assay. Percent suppression of cytokine production by Treg is calculated from the following equation: % Suppression = [1-(mean concentration Treg+Teff)/(mean concentration Teff alone) * 100]. Significant differences between SFM and 1%AS indicated as * $p<0.05$ and ** $p<0.01$.
