# Supplementary figures and images for: Influence of Membrane CD25 Stability on T Lymphocyte Activity: Implications for Immunoregulation
Source: PLoS One. 2009 Nov 24;4(11):e7980. doi: 10.1371/journal.pone.0007980 (PMC2775921; doi:10.1371/journal.pone.0007980)

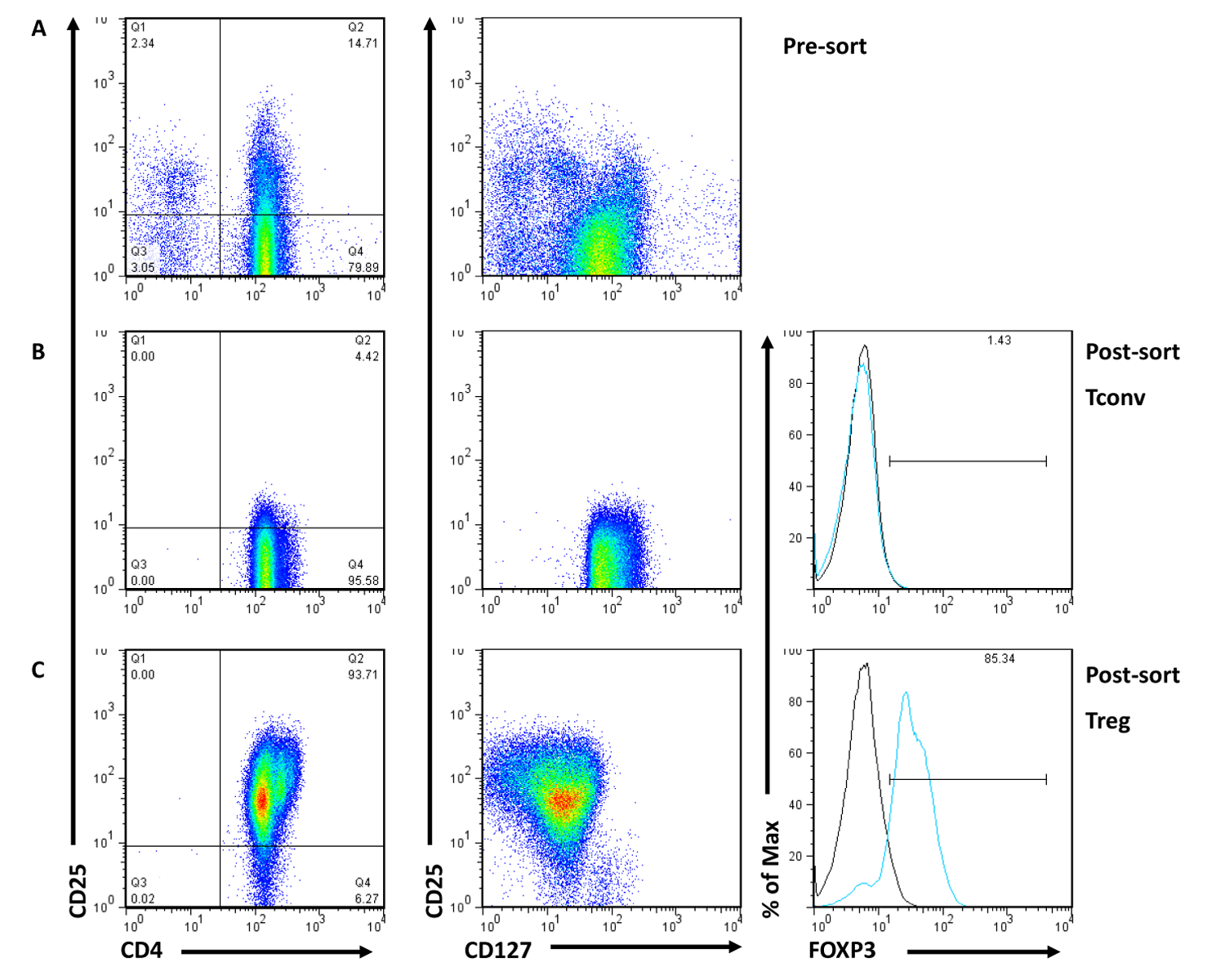

Supplement: Figure S1 — Isolation of Treg and T conventional cells from human peripheral blood by FACS. Representative plots showing (A) pre-sort expression profiles of CD4 (x-axis) and CD25 (y-axis, left plots) or CD25 (y-axis) and CD127 (x-axis, middle plots) following CD4 negative selection. Plots represent post-sort purity of sorted cells indicating expression of CD4, CD25, CD127, and FOXP3 for (B) T conventional cells and (C) Tregs. Post-sort FOXP3 analysis was assessed immediately following FACS isolation (right plots, black overlays represent isotype control and blue overlays indicate FOXP3 staining, respectively). (0.77 MB TIF) [file pone.0007980.s002.tif]

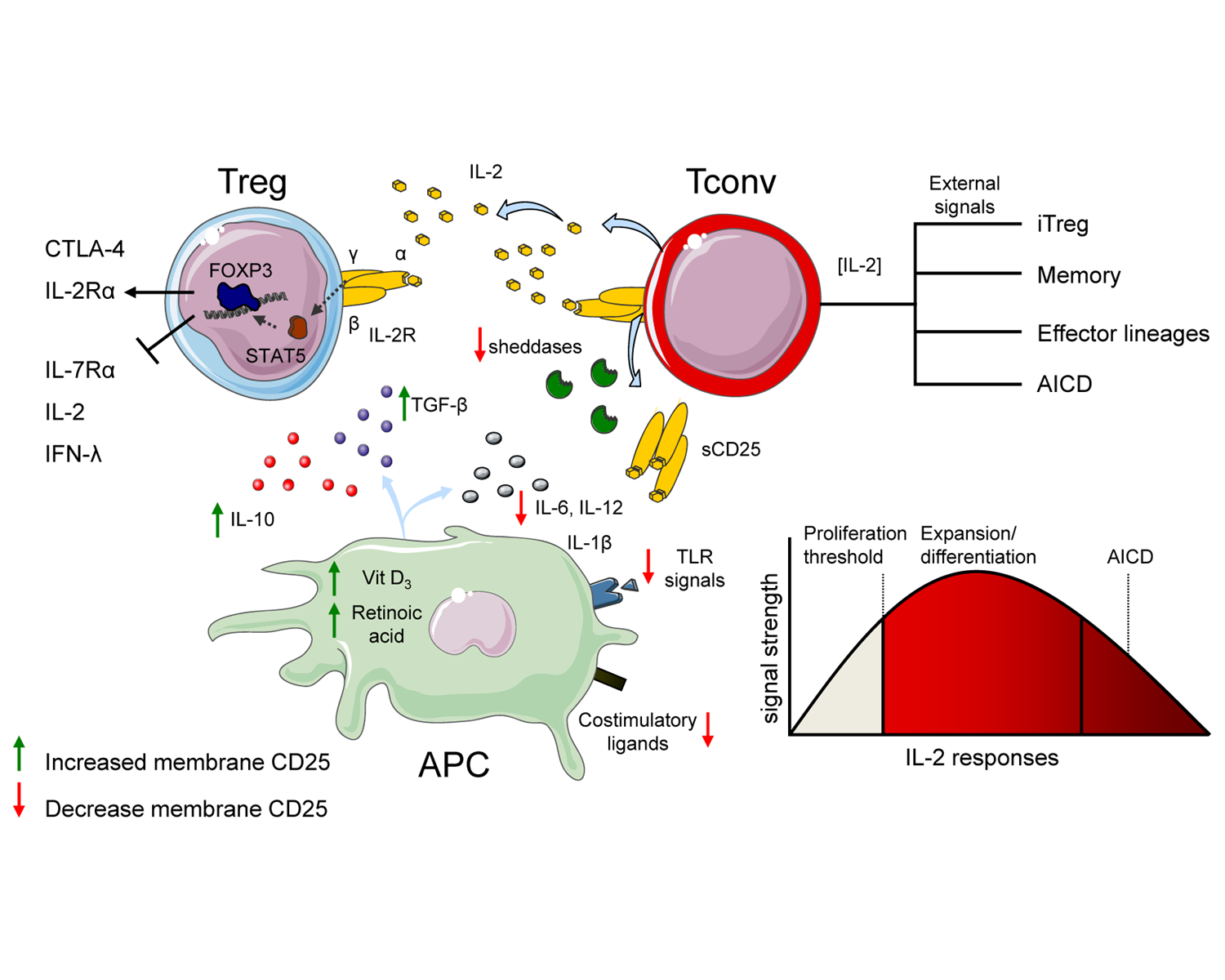

Supplement: Figure S2 — Membrane CD25 stability influences immune responsiveness. A hypothetical model is shown depicting how alterations in membrane-bound and soluble forms of CD25 (sCD25) may influence the activities of Treg and conventional T (Tconv) cell subsets. The immunological microenvironment provides costimulatory and cytokine signals through APC which influence CD25 stability on Treg and Tconv cells. Both intrinsic signals, through TCR signal strength and costimulation, as well as extrinsic inflammatory signals such as TOLL ligand induced proinflammatory cytokines may skew T cell phenotypes through the production of proteases and subsequent cleavage of membrane-bound CD25. Loss of membrane CD25 and IL-2 signaling in Treg would result in reduced phosphorylation of STAT5 and downstream FOXP3 expression. Conversely, in Tconv cell populations IL-2 signal strength along with costimulatory and cytokine differentiation signals would influence the lineage fate of responding T cell populations. Loss of CD25/IL-2 high affinity signaling from Teff cell populations would result in lower IL-2 affinity and/or dependence on signaling from other common γ-chain receptors for survival (such as IL-7, IL-15, and IL-21). Conversely, Tconv cells activated under tolerogenic conditions would lead to increased CD25 membrane stability and subsequent FOXP3 expression attenuating effector responses (Th17, Th1, and Th2) and reinforcing an induced Treg (iTreg) phenotype. The signal strength as measured by both affinity and avidity for IL-2 (related to the alpha beta gamma combinations) is important in driving proliferation but also AICD. (0.53 MB TIF) [file pone.0007980.s003.tif]
